# Psychological resilience in trench warfare: Leveraging mental health frameworks for Ukrainian soldiers

**DOI:** 10.1016/j.nsa.2025.105528

**Published:** 2025-09-30

**Authors:** Iryna Frankova, Vladyslav Klochkov, Marta Pyvovarenko, Oleh Hukovskyy, Joseph Zohar, Eric Vermetten

**Affiliations:** a Department of Clinical, Neuro- and Developmental Psychology, WHO Collaborating Center for Research and Dissemination of Psychological Interventions, Amsterdam Public Health Institute, Vrije Universiteit Amsterdam, Amsterdam, the Netherlands; b ARQ National Psychotrauma Centre, Centrum 45, Oegstgeest, the Netherlands; c Bogomolets National Medical University, Kyiv, Ukraine; d American University Kyiv, Kyiv, Ukraine; e Lesya Ukrainka Volyn National University, Lutsk, Ukraine; f Armed Forces of Ukraine, Ukraine; g Sheba Medical Centre, Tel HaShomer, Israel; h Leiden University Medical Center, Leiden, the Netherlands; i New York University, Department of Psychiatry, USA

**Keywords:** Combat stress, Continuous traumatic stress, Trauma, Military, War, Resilience

## Abstract

The ongoing Russia-Ukraine War has highlighted the profound psychological toll of asymmetrical and hybrid warfare on military personnel, underscoring the need for tailored mental health interventions. This opinion paper examines the psychological challenges faced by Ukrainian soldiers, including prolonged combat stress, moral injury, and exposure to traumatic events. It reviews the effectiveness and limitations of existing mental health frameworks, such as Combat and Operational Stress Control (COSC), and proposes adaptations for application in low- and middle-income contexts.

Key innovations in mental health support are explored, including the integration of digital tools, personalized interventions, and culturally adapted protocols like iCOVER, which emphasize resilience, flexibility, and coping efficacy. The paper also identifies gaps in training, preparedness, and long-term care, particularly in addressing continuous stressors like drone warfare, prolonged deployments, and hybrid psychological operations.

A revised framework is proposed to enhance psychological readiness, incorporating principles such as maintaining routines, fostering cohesion, and sustaining hope and purpose. These principles are vital for addressing the unique challenges of trench warfare and the psychological effects of protecting one's homeland. Insights from this study offer practical guidance for mental health professionals and policymakers, emphasizing the need for scalable, context-sensitive approaches to sustain the mental fitness and survivability of soldiers in protracted conflicts.

## 1 Introduction

The ongoing Russia-Ukraine War is largest conventional armed conflict in Europe since the World War II (Brands, 2024). Since Russia's full-scale invasion of Ukraine in 2022, the frontline stretches more than 3000 km, with 1000 km of active combat line (Shelest, 2024). Even though the current number of active military personnel on both sides exceeds one million, Russia asymmetrically possesses a tenfold superiority in terms of weaponry and firepower (Comparison of Russia and Ukraine, 2024). The mobilization of half a million civilians during the initial months of the full-scale invasion posed significant challenges to effective military preparation and training (Karachevskyy, 2023). The dynamics of warfare, characterized by intense combat and the introduction of novel weaponry, have given rise to new forms of mental maladjustment, stemming from insufficient military defense and a lack of relevant prior combat experience among recruits.

This war has not only reshaped the geopolitical landscape but has also illuminated the profound psychological toll that such warfare exacts on military. Ensuring the mental fitness and overall well-being of Ukrainian military personnel is of critical importance, as sustained physiological and psychological stressors can adversely affect motivation, and might cost lives of many (Hukovskyy et al., 2024). Service members deployed in frontline operations frequently encounter traumatic experiences, including combat situations, alongside persistent chronic stressors such as harsh field conditions, sleep deprivation, and high workloads (Morganstein et al., 2023). The cumulative impact of these stressors can lead to a range of psychological responses that pose significant risks to the well-being of the service member, their unit, and the overall effectiveness of their combat mission (Adler et al., 2022).

The psychological impact of combat and operational stress on military personnel has been a subject of extensive research, particularly in the context of modern warfare (Jetly, 2011) (Ursano et al., 2014) (Krauss et al., 2021). Various mental health programs and interventions have been developed and implemented by North Atlantic Treaty Organization (NATO) countries to address these mental health issues, with a specific focus on prevention frameworks aimed at mitigating the psychological sequelae of combat stress (Cooper et al., 2024). However, operational stress control (COSC) models must be adapted to reflect the realities of modern warfare, particularly in large-scale combat operations characterized by long-range precision munitions and advanced air defense systems, since these factors hinder access to and mobility within operational areas and disrupt support capabilities (Hoyt et al., 2021). The complexities of large-scale combat operations, particularly in the context of the war in Ukraine, necessitate a better understanding of the existing interventions’ adaptation and contextualization for soldiers, who are subjected to prolonged combat-related stress, and the challenges of operating in a chaotic and rapidly evolving environment.

This opinion paper examines the current body of knowledge regarding the mental health challenges faced by military personnel in the ongoing war in Ukraine. It highlights the principles underpinning immediate stress responses and presents evidence-based psychological interventions designed for preventive care during deployment. Additionally, it showcases adaptations of international COSC programs, tailored to align with the unique cultural and contextual needs of the local military framework. Finally, the paper advocates for a revision of the generally accepted principles of forward mental health care and explores the potential to align with the realities of modern warfare. This reevaluation is vital for preserving the mental resilience and operational effectiveness of combatants engaged in over three years of full-scale conflict in a low-to middle-income country, particularly in the absence of a unified consensus on best practices in this domain.

## 2 The psychological toll of asymmetric warfare and the need for innovation in PTSD prevention and treatment

Asymmetric warfare, characterized by a significant disparity in military capabilities and recourses, has been a defining feature of the armed conflict in Ukraine (Nilsson et al., 2021). The urgent pressure of war after the Russia's full-scale invasion necessitated the establishment of civilian units, commonly referred to as territorial defense units. Half a million civilians, lacking prior combat experience, were mobilized into military roles during the early stages in February 2022. This rapid mobilization has resulted in a lack of sufficient preparatory military training among combatants and lack of practical experience contributing to ineffective coping with the stressors of combat (Karachevskyy, 2023) (Hukovskyy et al., 2024). The prior combat experience and distinct military identity can be an important factor in the readiness and motivation of Ukrainian military personnel to participate in hostilities, however, there are no data published that would provide the insight into the level of readiness and adaptability of conscripts within last years (Rybinska et al., 2022).

The ongoing warfare in Ukraine has subjected military personnel to relentless artillery bombardments, the introduction of novel weaponry, including some that are illegal to use in warfare, successive offensives, and the constant challenge of distinguishing between adversarial and allied forces. These factors contribute to persistent and prolonged combat-related stress that undermines both the physical and psychological well-being of soldiers, ultimately compromising their survivability on the battlefield (Shelest, 2024) (Zabolonyi et al., 2023).

A significant development in the conflict has been the use of unmanned aerial vehicles (UAV) commonly known as drones, such as the Bayraktar TB2, a Turkish-made UAV, and Iranian-made kamikaze UAVs Shahed 131 and Shahed 136 that became one of the most impactful drones deployed in the war (Eslami, 2022). These UAVs have not only altered the tactical landscape but have also introduced a high-threat environment that intensifies psychological strain. In response to drone attacks, soldiers often report feelings of immobilization, described as being "glued to the ground," where the fear of imminent attack renders them unable to act or seek safety (Adler et al).

Phobia of drones reflects the psychological burden associated with advanced weaponry (Chávez, 2023). It highlights the need for mental health interventions that address not only traditional combat stressors but also the unique anxieties triggered by modern technological warfare.

The unique circumstances for the Ukrainian soldiers are that they are fighting and protecting homeland. An important component of the psychological readiness for risk of the Ukrainian military is their strong sense of patriotism, the ability to mobilize quickly, strong willpower, and positive experience of team cohesion (Prykhodko et al., 2021). Several factors have been identified as crucial to assure sustainment in high stress environment like warfare in Ukraine and determine the readiness of personnel to conduct combat operations (Kokun et al., 2022). The high morale associated with fighting on one's homeland could be also counterbalanced by the psychological toll of eye witnessing war crimes, crimes against civilians and mass casualties or poor military leadership. The battlefield conditions and warfare in Ukraine have introduced a range of new stress factors that significantly impact the mental health of soldiers (Box 1) (Neminsky et al., 2023). The interplay of these factors necessitates a nuanced understanding of the mental health challenges faced by soldiers in this context.

Box. 1 **Warfare stress factors in Ukraine.**

o ***Combat Conditions:*** Confinement in restricted spaces with limited mobility restrained daily activities
o ***Sleep Deprivation:*** Chronic lack of restorative sleep due to ongoing threats and operational demands.
o ***Social Isolation:*** Prolonged interaction with a single unit, with minimal contact with family or external support networks.
o ***Uncertainty in Deployment Duration:*** Extended rotations beyond one year, with unclear timelines for relief or return.
o ***Lack of Recovery Opportunities:*** Limited downtime between operations and insufficient resources for psychological recuperation.
o ***Weaponry Superiority of the Enemy:*** Persistent psychological strain from facing superior firepower and advanced technologies, such as unmanned aerial vehicles and long-range precision munitions.
o ***Psychological Pressure on Families:*** Targeted threats and misinformation campaigns directed at soldiers' families, exacerbating emotional burdens.
o ***Hybrid Warfare Tactics:*** Propaganda, disinformation, and cyberattacks undermining morale and cohesion.
o ***Witnessing Destruction and Atrocities:*** Exposure to civilian casualties, mass destruction, and war crimes leading to moral injury.
o ***Environmental Challenges:*** Harsh weather conditions and inadequate shelter impacting physical and mental well-being

Alt-text: Box. 1

War produces different kinds of long-lasting consequences, such as damage to infrastructure, economic hardship, and loss of health and life. Another important factor that makes this situation unique is ongoing complex emergency situation on the country level, the whole country is a target of military attack on a daily basis for over 1000 days. The primary targets of these attacks are not just military objects, but critical civilian infrastructure, manufacturing facilities, storage companies, as well as residential areas, hospitals or hotels suspected of harboring combatants (Frankova et al., 2024). The spring of 2024 saw a significant surge in attacks against the energy infrastructure, resulting in a major blackout during summer and a shortage of heating in autumn. The Ukrainian population and Ukrainian soldiers are witnessing the devastation of their homeland and exposed to continuous traumatic stress (Frankova et al., 2025). They are experiencing not just profound personal losses, on top of that they are burdened by concerns for the well-being of their families and communities, adding an additional layer of psychological burden. Moreover, the enemy puts psychological pressure on combatant's family via phone threats and other forms of mis- and disinformation as part of psychological operations (Vermetten et al., 2020). Another significant threat is captivity. The exact number of combatants who are missing in action or currently held captive remains unknown. Since the beginning of 2014, and up to July 1, 2019, a total of 3245 individuals have been located and released from captivity. Released prisoners of war have reported that the cessation of resistance, intimidation, displays of power, and the extraction of intelligence were the primary reasons for their capture (Timchenko et al., 2021). Hybrid attacks often target not just physical, but psychological, cultural, political, and human dimensions. They also resort to aggressive propaganda in social media at the local and international levels and various types of cyber warfare, economic blackmail, and diplomatic manipulations (Hoffman, 2009).

## 3 Neuroscientific insights into resilience and adaptation during combat stress

All war related stressors entail number of common characteristics, including uncertainty, unpredictability, major unexpected and inescapable life changes, a range of losses (loss of lives, safety, belonging, social standing and status, food security) and exposure to horrific scenes of death and destruction (Shalev, 2022). Research has demonstrated that exposure to war and combat-related stress can result in a range of mental health sequelae, like PTSD, depression, anxiety, substance abuse (Kozariæ-Kovaèiæ et al., 2001). However, in a majority of trauma-exposed individuals, the most common trajectory after exposure to traumatic experience is a healthy functioning, low level of symptoms or resilience, observed on average of two-thirds (66 %) of participants across numerous studies (Bonanno, 2008), (Galatzer et al., 2018), including the context of combat deployment of military personnel (Andersen et al., 2014), (Bonanno et al., 2012), (Donoho et al., 2017). The majority of individuals exposed to combat found out the right behavior or strategy that align with the specific context and timing. Given that warfare entails continuous exposure to a variety of stressors, it is essential to consider not only the responses to these stressors across multiple bio-psycho-social levels but also the dynamics of these responses over time. Therefore, the concept of resilience, coping efficacy and flexible self-regulation are particularly relevant in the context of combat stress.

Resilience operates on multiple levels, from cognitive and emotional processes including problem-solving and emotional regulation to neurobiological mechanisms like stress hormone regulation and neural plasticity (Pasteuning et al., 2024). Neural plasticity is increasingly acknowledged as a fundamental process in mental health, as it enables the reorganization of neural circuits and behavioral patterns essential for adapting to life's changing environment (Price et al., 2020). The increase in an individual's flexibility, capacity to adapt to new circumstances, or learning of new coping mechanisms, and recovering from psychological distress, is a manifestation of neural plasticity per se (Lövdén et al., 2010).

Charles Burton and George Bonanno developed a flexibility model, for example, consisting of three consequently related component abilities (Bonanno et al., 2013). This model provides a practical framework for understanding how soldiers can navigate the complexities of combat-related stress by leveraging adaptive mechanisms at critical decision-making junctures. The initial stage, Context Sensitivity, describes the ability to accurately decode situational cues so as to set a goal for subsequent regulation. Next, the Repertoire stage involves selecting a regulatory strategy to meet that goal from one's personal toolbox of strategies. Finally, during the Feedback stage individuals monitor the efficacy of the chosen strategy and, if needed, modify or change to a new strategy. Arieh Y. Shalev emphasizes the need for sustained responsiveness and adaptability in soldiers facing ongoing life-threatening situations (Shalev, 2022). Therefore, interventions targeting combatants survivability must enhance coping efficacy, enabling soldiers to maintain stable performance, emotional control, and meaningful relationships while remaining flexible in their decision-making processes.

Resilience research represents transitioning from a disease-centric approach to one that emphasizes health promotion and focuses on exploration of the mechanisms that can protect individuals against stress-related illnesses (Kalisch et al., 2017). Understanding the neuroscientific underpinnings of stress and resilience is essential for developing effective mental health interventions in combat settings lacking certainty regarding the duration of deployment and full recovery opportunities. Incorporating regulatory flexibility into resilience training offers a practical pathway for developing interventions that address the unpredictable and high-stakes environment of combat. By equipping soldiers with the tools to accurately assess, adapt, and refine their responses, mental health interventions can foster sustainable coping mechanisms. Such approaches can mitigate the cumulative effects of combat stress, promoting mental fitness and operational readiness in even the most challenging environments.

## 4 Prevention and combat and operational stress control interventions

Strengthening strategies mitigate the risk by preparing for potentially traumatic events and responding to the needs of those who may become affected. Forward mental health core elements include prevention (proper selection, mental fitness and training), combat stress identification, mitigation and follow up during and after operation and clinic-based treatment and timely short term recovery (NATO, 2019) (Mamedov et al., 2024). A number of mental health programs and interventions were developed to address the psychological sequelae of combat and operational stress through far-forward intervention on the battlefield (Cooper et al., 2024). Such interventions, broadly conceptualized as Combat and Operational Stress Control (COSC), seek to mitigate emerging symptoms in the immediate aftermath of combat and reduce the negative effects of prolonged operations in austere environments (Brusher, 2007). The Proximity, Immediacy, Expectancy, Simplicity (PIES) principles, which serve as a great example of an immediate response to operational stress after combat missions aimed at preventing post-traumatic stress disorder (PTSD) and quickly returning soldiers to combat, are utilized by military services within the United States and other NATO countries (West et al., 2019).

Core principles include a) mental health support provided as close to casualty unit as possible (*Proximity*); b) as soon as possible (*Immediacy*); c) with the *Expectation* that they will return to their unit fully ‘fit to fight’; d) with the minimum intervention possible (*Simplicity*) (NATO, 2019). Lately United States military services use PIES with some modification of that principles BICEPS to include *Brevity* (provide brief treatment for rapid return to duty) and *Contact/Centrality* (direct involvement by unit leaders in the service member's recovery) (True et al., 1992).

COSC programs have significant potential to enhance operational readiness by emphasizing return to duty and by decreasing the number of service members who must be medically evacuated due to psychological reasons (Rapley et al., 2017). Currently, the Psychological Support for Personnel (PSP) also known in past as Moral and Psychological Support (MPS) in the Armed Forces of Ukraine is responsible for monitoring and promoting the psychosocial well-being of active service members during deployment. The rapid and substantial increase in the size of the Ukrainian Army has led to a heightened demand for psychological support among military personnel, thereby making the role of military psychologists increasingly vital. The PSP personnel comprise 15,000 psychological officers and 2000 military social workers (Neminsky et al., 2023). In 2021, the Armed Forces of Ukraine initiated the integration of "combat stress control groups" within select brigades, incorporating COSC principles into unit practices (Hukovskyy et al., 2024). The number of personnel in combat stress control and psychological support and recovery groups has increased from 250 to 2000 psychologists. However, the goal is to expand this workforce to 5000 staff members.

Recent scoping review has identified 36 reports on 19 different COSC programs aligning them with consideration of methodological levels of evidence and in accordance with prevention framework, that entails three levels of prevention, universal, selective and indicated (Cooper et al., 2024). The first level universal prevention programs are targeting all military population. Second, selective prevention programs are developed for deployed service members (at risk) and include programs, implemented in four different settings: (a) prior to deployment; (b) in operational training environments; (c) during deployment; and (d) in a post-deployment. And lastly, indicated prevention programs are implemented in the early stages within deployment settings via clinical treatment for the military who experience mental health issues. Eleven of the 19 COSC programs had been evaluated using comparison conditions or control groups, predominantly those were second level of prevention interventions. Following selective prevention programs for different settings (pre-, during, post-deployment) were tested in randomized controlled trials: pre-deployment stress inoculation training (Hourani et al., 2016) (Hourani et al., 2011) (Hourani et al., 2018), technology enhanced relaxation (Stetz et al., 2011), mindfulness-based mind fit training (Jha et al., 2017) (Jha et al., 2010) (Johnson et al., 2014) skills training prior to survival course (Taylor et al., 2011), iCOVER (Adler et al., 2020), yoga warrior (Stoller et al., 2012), event-driven psychological debriefing (Adler et al., 2008) (MacDonald, 2003) (Pischke et al., 2008) and battlemind training (Adler et al., 2011) (Castro et al., 2012). Indeed barriers precluding clinical trials in combat and operational settings are the inability to randomize patients seeking care to treatment conditions and the difficulties following patients longitudinally once they return from deployment.

Recent international study suggests that up to 52 % combatants report acute stress reactions (ASR) in military operations that last more than 5 min, with 19 % reporting that the impairment lasted more than a day (Adler et al., 2022). These estimates underscore the importance of intervening immediately in order to prevent the military unit from being unable to perform because of reduced capability of its team members. Integrating mental health support into military training is essential for ensuring that soldiers are adequately prepared to cope with combat stresses, enhance resilience, and improve overall performance (NATO, 2019). In 2014, the Israel Defense Force developed and implemented a peer-based intervention called YaHaLOM to address ARS in unit members (Svetlitzky et al., 2020). Later, this protocol was adapted and tailored for different contexts by five countries (Canada, Germany, Norway, the UK, and the USA) and renamed iCOVER while maintaining its core principles. iCOVER stands for Identify, Connect, Offer commitment, Verify facts, Establish order of events, Request action (Adler et al., 2024a). As part of international collaborative approach to ASR management iCOVER, was translated and adapted by the Norwegian Armed Forces using the acronym ReSTART. Recently, this collaborative effort has been extended to the Armed Forces of Ukraine (AFU) through, among other initiatives, Norway's training of Ukrainian combat medics (Iversen et al., 2023). The iCOVER protocol, is already translated into Ukrainian, and the training materials were culturally adapted with emphasis on the importance of rituals, additional module on grief, on mental skills for handling human remains (Nordstrand et al., 2024). iCOVER protocol it is not yet part of the formal AFU pre-deployment training, but informally is used in some brigades as an important supplement in preparing newly conscripted troops like those fighting in Ukraine. The use of iCOVER during the drone attacks was reported to help overcome ASR, enabling combatants to reach a safer location. The 120 min training covers education on the neural basis of ASRs and how to recognize it, introduction to the six iCOVER steps, how to deal with non-responders, and how to implement the procedure in a combat context. The training is conducted using a standardized package of training materials, cue- and instruction cards, standardized PowerPoint slides, and an instructional video (Nordstrand et al., 2024). Results demonstrate positive perceptions, acceptance and increased confidence in ability to manage ARS and self-care among Ukrainian combat medics from before to after training (Adler et al.), (Nordstrand et al.). Some efforts in Armed Forces of Ukraine focus on prevention and pre-deployment training. One case example reports that elements of resilience and psychological preparedness were integrated into the shooting training of Ukrainian border guard officers, acknowledging the impact of those elements on effective performance during combat (Zabolonyi et al., 2023).

## 5 Discussion, future directions for advancing mental health support in warfare

Even though COSC concepts are well-known and well-established, in the immediate ongoing (intratraumatic) stress situation like full scale invasion of Russia and ongoing war in Ukraine, they are not sufficient due to unique circumstances of large scale combat operations at the theatre.

The core principles of COSC were developed by military allies and demonstrated its effectiveness in external military operations, with defined deployment period of 6–9 months. When military personnel deployed elsewhere, their homeland, families and loved ones remain safe. Additionally, government health care and social systems, and funding opportunities for post-combat recovery stay untouched and play a crucial role in the well-being of deployed personnel and their families. Whereas, combatants at the frontline in Ukraine face confined living conditions, significant space restrictions, and limited daily activities. Soldiers experience sleep deprivation and are often isolated with limited contact with family. Additionally, uncertainty about the rotation, due to prolonged deployments in a situation of manpower shortage undermine military morale. Military conflicts arising from invasion can result in a tenfold increase in hospitalizations of military personnel in psychiatric facilities. Among these inpatients, the most prevalent mental health issues include anxiety disorders, dissociative disorders, stress-related disorders, somatoform disorders, and other non-psychotic conditions (Druz et al., 2025). Since February 2022, the World Health Organization has confirmed over 325 incidents of attacks on healthcare facilities within Ukraine. Furthermore, more than 800 healthcare institutions have sustained damage, with over 120 of these facilities rendered irreparable (Ukraine et al., 2023). The ongoing conflict has precipitated significant economic and social repercussions for Ukraine, leading to the disruption of health services for both military personnel and veterans' health programs (Frankova et al., 2024). Nevertheless, the high prevalence of stress-related disorders among military personnel in wartime situations necessitates the rapid implementation of scalable interventions focused on appropriate management and, especially, prevention (Druz et al., 2025).

### 5.1 Revised core principles of psychological support in military contexts

The psychological toll of prolonged exposure to traumatic stress underscores the need to expand upon the foundational principles of psychological support in military contexts. To address these evolving challenges, the following updated framework is proposed (Box 2).

Box 2 **Revised core principles of psychological support in military contexts.**

o ***Maintain discipline and routine****:* Establish habits to manage daily stress effectively, with particular attention to maintaining consistent sleep routines.
o ***Reinforce cohesion and assure proximity****:* Strengthen social bonds among peers and ensure individuals remain close to their units and the front lines to foster a sense of unity and shared purpose.
o ***Promote social support****:* Actively engage family, friends, and colleagues to provide a reliable network of emotional and practical assistance.
o ***Foster hope****:* Cultivate forward-looking perspectives and encourage metacognitive strategies to maintain optimism about the future.
o ***Enhance sense of purpose and identity***: Facilitate meaning-making by addressing fundamental questions such as "Why are we here?" and "What is our mission?" while supporting the development of personal and collective identity.

Alt-text: Box 2

First three layers are well known, integrated into existing COSC programs, and well accepted by local and international military psychology experts. Recently effective strategies for promoting psychological health in high-stress environments were nicely summarized (Adler et al., 2024b). They strongly advocate for sleep prioritization as a vital aspect of recovery. Military leaders should emphasize the importance of sleep while also integrating mental skills like psychological grounding and deep breathing into daily routines to bolster individual resilience. Additionally, they emphasized the importance of managing anger through emotion regulation techniques is crucial, as unchecked anger can hinder communication and functioning. Overall, reinforcing these stress-mitigating strategies can improve mental health trajectories and military readiness, ensuring they are not overlooked amid mission-critical tasks.

However forth and the fifth layers are not explicitly mentioned in the existing COSC principles, yet they have proven to be important in Ukraine facing adversities of war. One real practice example among many describes the adaptation of widely used by international military community COSC program during the deployment called time-driven psychological debriefing to the Combat Path Debriefing adding those concepts to the core of the protocol. This facilitated 90 min group discussion pays significant attention to the unit level, it's history, unique narrative and path, emphasizing the significance of a military identity, acknowledging grief and enhancing sense of purpose, introducing simple stress-reduction techniques and strengthen unit cohesion (Hukovskyy et al., 2024). Morale is the crucial component of fighter's spirit. Efficient brigades in Ukraine invest a lot of efforts into the formation of national identity, unit identity, and cultural values of a warrior, military rituals.

The revised core principles do not exclude or replace the important work already accomplished in the integration and implementation of evidence-based Combat Operational Stress Control (COSC) pre-deployment training programs that are tailored to local contexts and cultures. Recently, a comprehensive approach to forward mental health intervention has been proposed to address the challenges associated with large-scale combat operations (Hoyt et al., 2021). This approach includes enhanced training and utilization of self-aid and buddy aid, establishing a baseline of training in forward behavioral health intervention for medics and battalion-level medical providers, and increasing the involvement of paraprofessionals and chaplains in forward settings. While empirically supported COSC programs can generally be implemented in new settings without the need for additional validation, several crucial aspects must be considered: a) cultural and contextual adaptation is essential; b) the selection of programs should be driven by evidence and neuroscience, while also recognizing the importance of flexibility and coping efficacy; c) military leaders must identify systematic methods for collecting clinical data in forward clinics and other COSC settings as part of routine care.

Several other extra considerations seem to be important in the context of conventional warfare, mental fitness and survivability.

### 5.2 Leadership

Psychological resilience in the context of war is the ability of not just individuals, but teams, and military units to adapt and recover in situations of risk, challenge, and life-threat (Klochkov, 2022) (Litz et al., 2009). For teams, fostering cohesion and maintaining a clear sense of purpose can enhance collective resilience, especially during warfare in Ukraine (Boyko, 2017). Team cohesion and leaders are critical for maintaining resilience and positive mental health trajectories (Crane et al., 2022). Leader emphasis on the team's purpose and values, especially in a state of uncertainty or setback, are crucial in sustaining optimism and commitment (Adler et al., 2024b).

What leaders do and say plays a critical role in protecting the mental health and readiness of military. Daily communication of military leaders and chains of command with their subordinate personnel can promote hope, create and sustain meaning, and foster a sense of purpose that contributes to persistent resilience. The evaluation of the leadership qualities of commanders is defined by the Doctrine of Military Leadership in the Armed Forces of Ukraine and is conducted using the "360° Assessment" ("Otsinka 360°) tool, which reflects feedback from a group of respondents including superiors, colleagues, and subordinates. Unfortunately, Ukrainian military command might underestimate the significance of leadership in the unit's psychological resilience. The relationship between leader and combatants within the unit, confidence in the commander, trust to the chain of command, and his competence in addressing mental health needs are crucial in the sustainment of survivability of the combatants. To address this issue systematically, combat an operational stress control guidance is being developed for command chains. However, there are cultural challenges to consider. High levels of distrust toward authorities in Ukrainian culture are rooted in the legacies of past Soviet regime (Gorbunova et al., 2020).

### 5.3 Ethical considerations in combat mental health

Military operations often involve moral dilemmas and difficult decisions that affect the well-being of the decision-makers, their subordinates and peers, their adversaries and civilian non-combatants (Vermetten et al., 2018). The difficulty stems from the fact that these decisions may require the service member to choose between mission success, civilian safety and military force protection. The decision-making process concerning the return of personnel with acute stress reactions and other mental health issues to duty to their units is fraught with ethical considerations, particularly in scenarios where the absence of service members may compromise unit safety and operational effectiveness. For instance, when evaluating whether to permit an individual with a mental health casualty to remain in the unit and return to combat or sent out for further diagnostic and treatment the determining factor often revolves around whether such a decision would safeguard the welfare of the remaining unit members. The challenge and potentially moral dilemma lie in establishing appropriate boundaries and guidelines for return to duties, treatment and rehabilitation duration, particularly in contexts of long-duration warfare where personnel shortages may necessitate difficult choices.

Military operations involve decision-making at every level (strategic through tactical) and these decisions can have important moral implications for military personnel at all levels. Moral injury has been defined as “perpetrating, failing to prevent, bearing witness to, or learning about acts that transgress deeply held moral beliefs and expectations” (Litz et al., 2009). Moral injury among the Armed Forces of Ukraine is operationally defined as a form of mental suffering, a decline in mental health, and the humiliation of honour and dignity that arises from the actions or inactions of specific officials, such as unlawful orders, violations of rights, unjust treatment, breaches of statutory norms, combat injuries, and the loss of comrades. This is especially relevant for nowadays Ukraine due to complexities associated with distinguishing between adversarial and allied forces and risk of ‘friendly fire’. Several mechanisms for legal protection are available, including appeals to the military prosecutor's office or military law enforcement agencies, as well as submitting complaints through the chain of command to the Commissioner of the Verkhovna Rada for Human Rights. However, despite the existing system, several systemic issues persist. These include a lack of awareness among military personnel regarding their legal rights and delays in judicial and compensation processes. However, the state of the science has progressed to the point where there is much that national militaries can do to prepare their service members developing and moral resilience training programs (Rushton, 2017). (Barth et al., 2020), (Georgoulas et al., 2022). For instance, psychological training designed to prepare military personnel of the Armed Forces of Ukraine for the demands of captivity (forced detention) has been developed in accordance with NATO standard STANAG 7226. This training is integrated into the basic military curriculum, focusing on enhancing moral and psychological resilience as well as survivability in captivity (Neminsky et al., 2023). The role of spiritual support and military chaplains constitute a special role in the moral resilience of soldiers experiencing moral stress (Schuhmann et al., 2023).

### 5.4 Multisectoral approaches to mental health support

One of the approaches to overcome the gap between the need and capacity to provide mental health support to active-duty military would be to engage civilians and build a capacity in different sectors, like medical service, social policy, etc. In current situation military personnel already assisted by civilian doctors within the framework of a unified medical space (Karachevskyy, 2023). Recently institutional and legal foundations for such adjustments were assessed and proved to be feasible, respected recommendations were given (Karamyshev et al., 2022). Proper medical support system of the Armed Forces of Ukraine based on the effective and rational use of medical resources within the framework of a unified medical space creating is crucial, since the funding for mental health within military traditionally is limited.

### 5.5 Innovations and digital solutions implementation

Digital technologies and digital mental health interventions serve as alternative to the existing task-shifting and peer based in person interventions and called to address the gap between the number of affected individuals and capacity to address their mental health needs. The advantage of digital interventions lies primarily in their cost and recourse effectiveness, versatility, and accessibility (Andersson et al., 2018). Implementation of digital interventions into the maintenance of the psychological health and resilience of military personnel might potentially address a number of challenges, that armed forces are facing during the war and has a number of advantages for military personnel a) flexible timing of assistance; b) addressing the stigma and shame to ask for support; c) complete confidentiality; d) more accurate assessment of the current emotional state; minimization of negative attitudes towards interventions in the mental health sphere; e) absence of human factor; f) possibility to collect information remotely (Van Voorhees, 2012). There are also barriers to the utilization of digital mental health tools in field settings, such as frequent signal disruptions and the threat posed by precision munitions, which compromise signal reliability. However, mobile self-help applications that incorporate data-at-rest encryption, are pre-installed on devices, and operate without requiring internet connectivity have the potential to overcome these obstacles (Hoyt et al., 2021). Considering large number of military personnel joined the armed forces of Ukraine, known shortage of mental health staff capable to deliver timely support, logistical barriers to receive support and high levels of stigma there is a need to design and assess feasibility of digital mental health tool to support preparedness of the personnel to adapt to and overcome high-stress combat environments.

## 6 Conclusion

The literature on combat and operational stress control underscores the necessity for ongoing research and adaptation of mental health interventions in military contexts, particularly in response to the unique challenges posed by modern warfare. The ongoing conflict in Ukraine emphasizes the importance of scalable, multisectoral approaches, including the integration of civilian health professionals and the adaptation of established mental health frameworks to meet the needs of soldiers exposed to continuous combat-related stress. Future research must continue exploring innovative strategies to enhance the psychological resilience of military personnel, ensuring that they are equipped to navigate the multifaceted challenges of combat and operational stress effectively. Additionally, it is essential to continuously update training materials and combat stress-control protocols to address emerging threats posed by new weaponry and the hybrid nature of warfare.

The unique stressors faced by Ukrainian soldiers, such as the witnessing of destruction and personal losses, further complicate their psychological resilience. The need for tailored mental health interventions that address these specific stressors is paramount to sustaining the survivability and mental fitness of combatants in this context. Incorporating the concept of regulatory flexibility into these interventions offers a promising avenue for enhancing soldiers' adaptability in dynamic combat environments. Regulatory flexibility provides a framework for understanding how soldiers can navigate uncertainty by accurately assessing situational cues (context sensitivity), employing diverse coping strategies (repertoire development), and adjusting approaches as needed (feedback). Interventions that teach and reinforce these adaptive processes could empower soldiers to maintain stable performance and emotional control under continuous stress.

To meet these challenges, several key considerations must guide future efforts in forward mental health care: mental health support must be adapted to the scale of the conflict and economic constraints:

● Scaling training: Effective preparation for large number of civilians and military personnel requires scalable and culturally relevant training frameworks.
● Digital interventions: while promising in terms of accessibility and scalability, digital solutions must address security and logistical challenges in conflict zones.
● Moral injury and prolonged stress: The mitigation of psychological impact of moral injury and continuous traumatic exposure must be central to intervention strategies
● Sustaining hope, trust, and purpose: Long term mental fitness requires interventions that foster a sense of purpose, maintain trust within units, and instill hope, even under the harshest conditions.

## Authors note

The opinions expressed in this paper are those of the authors and do not necessarily represent the decisions, policies, or views of the organizations they serve or the funder. The funders are not liable for any use that may be made of the information contained therein. The authors have no conflicts of interest to declare. Data summarized in the panel resulted from studies that had approval from the relevant ethics boards.

## Contributors

All authors contributed to the conceptualization, writing – original draft, writing – review & editing. All authors read and approved the final manuscript.

## Ethics Statement

N/A.

## Role of funding source

This research did not receive any specific grant funding agencies in the public, commercial, or not-for-profit sectors.

## Declaration of competing interest

The authors declare that they have no known competing financial interests or personal relationships that could have appeared to influence the work reported in this paper.

## References

[bib1] Adler A.B., Nordstrand Andreas Espetvedt, Engen Haakon Gabrielsen, Vermetten E. (2025). Digital Tools for Prevention, Mitigation, and Treatment of Traumatic Stress Responses in Terror and War - Contemporary Practices and Novel Insights.

[bib2] Adler A.B. (2008). A group randomized trial of critical incident stress debriefing provided to US peacekeepers. J. Trauma Stress: Off. Publ. Int. Soc. Traumat. Stress Stud..

[bib3] Adler A.B. (2011).

[bib4] Adler A.B. (2020). Rapid response to acute stress reaction: pilot test of iCOVER training for military units. Psychological Trauma: Theor. Res. Pract. Policy.

[bib5] Adler A.B., Gutierrez I.A. (2022). Acute stress reaction in combat: emerging evidence and peer-based interventions. Curr. Psychiatry Rep..

[bib6] Adler A.B. (2024). Peer-based intervention for acute stress reaction: adaptations by five militaries. BMJ Mil. Health.

[bib7] Adler A.B., Forbes D., Ursano R. (2024). Sustaining NATO service member mental health during the crisis in Ukraine. BMJ Mil. Health.

[bib8] Andersen S.B. (2014). Latent trajectories of trauma symptoms and resilience: the 3-year longitudinal prospective USPER study of Danish veterans deployed in Afghanistan. J. Clin. Psychiatr..

[bib9] Andersson E., Holmes E.A., Kavanagh D. (2018). Innovations in digital interventions for psychological trauma: harnessing advances in cognitive science. mHealth.

[bib10] Barth T.M. (2020).

[bib11] Bonanno G.A. (2008).

[bib12] Bonanno G.A. (2012). Trajectories of trauma symptoms and resilience in deployed US military service members: prospective cohort study. Br. J. Psychiatr..

[bib13] Bonanno G.A., Burton C.L. (2013). Regulatory flexibility: an individual differences perspective on coping and emotion regulation. Perspect. Psychol. Sci..

[bib14] Boyko O. (2017). The value-based concept of developing leadership competence in future officers of the armed forces of Ukraine in higher military educational institutions. Milit. Educ..

[bib15] Brands H. (2024). War in Ukraine: Conflict, Strategy, and the Return of a Fractured World.

[bib16] Brusher E.A. (2007). Combat and operational stress control. Int. J. Emerg. Ment. Health.

[bib17] Castro C.A. (2012). Mental health training with soldiers four months after returning from Iraq: randomization by platoon. J. Trauma Stress.

[bib18] Chávez K. (2023). Learning on the fly. Arms Control Today.

[bib19] (2024). Comparison of Russia and Ukraine military strengths. https://www.globalfirepower.com/countries-comparison-detail.php?country1=russia&country2=ukraine.

[bib20] Cooper D.C. (2024). Combat and operational stress programs and interventions: a scoping review using a tiered prevention framework. Mil. Psychol..

[bib21] Crane M.F. (2022). The interplay between social interaction quality and wellbeing in military personnel during their initial two-years of service. Mil. Psychol..

[bib22] Donoho C.J. (2017). A decade of war: prospective trajectories of posttraumatic stress disorder symptoms among deployed US military personnel and the influence of combat exposure. Am. J. Epidemiol..

[bib23] Druz O., Chaban O., Frankova I., Lahutina S., Lyzak O., Kyryliuk S., Khaustova O. (2025). Prevalence and distribution of mental disorders at inpatient psychiatry service of a large military hospital in Ukraine. Psychiatr. Clin Psychopharmacol..

[bib24] Eslami M. (2022). Iran's drone supply to Russia and changing dynamics of the Ukraine war. J. Peace Nucl. Disarm..

[bib25] Frankova I. (2024).

[bib26] Frankova I., Senyk O., Avramchuk O., Leshchuk I., Rudys A., Kurapov A., Goral A. (2025). Psychometric properties of the revised Ukrainian version of the Continuous traumatic Stress response scale (CTSR) in the context of the russo-ukrainian war. Eur. J. Psychotraumatol..

[bib27] Galatzer-Levy I.R., Huang S.H., Bonanno G.A. (2018). Trajectories of resilience and dysfunction following potential trauma: a review and statistical evaluation. Clin. Psychol. Rev..

[bib28] Georgoulas-Sherry V., Hernandez H.G. (2022). The effects of grit and resilience on moral competence following simulated combat exposure. Mil. Psychol..

[bib29] Gorbunova V., Klymchuk V. (2020). The psychological consequences of the holodomor in Ukraine. East W.: J. Ukrainian Stud..

[bib30] Hoffman F.G. (2009).

[bib31] Hourani L.L. (2011). Predeployment stress inoculation training for primary prevention of combat-related stress disorders. J. Cyberther. Rehabil..

[bib32] Hourani L. (2016). Toward preventing post-traumatic stress disorder: development and testing of a pilot predeployment stress inoculation training program. Mil. Med..

[bib33] Hourani L. (2018). Effect of stress inoculation training with relaxation breathing on perceived stress and posttraumatic stress disorder in the military: a longitudinal study. Int. J. Stress Manag..

[bib34] Hoyt T., Hein C. (2021). Combat and operational stress control in the prolonged field care environment. Mil. Rev..

[bib35] Hukovskyy O. (2024). The combat path: sustaining mental readiness in Ukrainian soldiers. US Army War College Quart.: Paramet..

[bib36] Iversen P., Adler A.B. (2023). Norway partner to help stressed Ukrainian troops. ARMY Magaz..

[bib37] Jetly C.R. (2011). Psychiatric lessons learned in Kandahar. Can. J. Surg..

[bib38] Jha A.P. (2010). Examining the protective effects of mindfulness training on working memory capacity and affective experience. Emotion.

[bib39] Jha A.P. (2017). Practice is protective: mindfulness training promotes cognitive resilience in high-stress cohorts. Mindfulness.

[bib40] Johnson D.C. (2014). Modifying resilience mechanisms in at-risk individuals: a controlled study of mindfulness training in marines preparing for deployment. Am. J. Psychiatr..

[bib41] Kalisch R. (2017). The resilience framework as a strategy to combat stress-related disorders. Nat. Hum. Behav..

[bib42] Karachevskyy A. (2023). Mental health care for Ukrainian military personnel during the war. Psychosomat. Med. General Pract..

[bib43] Karamyshev D. (2022).

[bib44] Klochkov V.V. (2022). Peculiarities in the development of psychological resilience among future officers of the armed forces of Ukraine. Bullet. Nat. Univer. Def. Ukraine.

[bib45] Kokun O.M., Klochkov V.V., Moroz V.M., Pishko I.O., Lozinska N.S. (2022).

[bib46] Kozariæ-Kovaèiæ D., Hercigonja D.K., Grubišiæ-Iliæ M. (2001). Posttraumatic stress disorder and depression in soldiers with combat experiences. Croat. Med. J..

[bib47] Krauss S.W. (2021). Distinguishing the effects of life threat, killing enemy combatants, and unjust war events in US service members. J. Trauma Stress.

[bib48] Litz B.T. (2009). Moral injury and moral repair in war veterans: a preliminary model and intervention strategy. Clin. Psychol. Rev..

[bib49] Lövdén M. (2010). A theoretical framework for the study of adult cognitive plasticity. Psychol. Bull..

[bib50] MacDonald C.M. (2003). Evaluation of stress debriefing interventions with military populations. Mil. Med..

[bib51] Mamedov R.C., Moroz V.M., Oliynyk V.O., Santashov V.I., Koval M.A., Mas’ N.M., Klochkov V.V. (2024).

[bib52] Morganstein J.C., Bromet E.J., Shigemura J. (2023). The neuropsychiatric aftermath of exposure to weapons of mass destruction: applying historical lessons to protect health during the war in Ukraine. Psychol. Med..

[bib53] NATO (2019). STANAG 2564.

[bib54] Neminsky I., Roy O., Romanyshin A., Olenchuk V., Pshenychniuk G., Hyzhnyak M., Kravchenko K., Kvych S., Hoptiy O., Klochkov V. (2023). Scientific and Research Centre of Humanitarian Issues of the Armed Forces of Ukraine.

[bib55] Nilsson N. (2021).

[bib56] Nordstrand A.E. (2025).

[bib57] Nordstrand A.E. (2024). A novel intervention for acute stress reaction: exploring the feasibility of ReSTART among Norwegian soldiers. Eur. J. Psychotraumatol..

[bib58] Pasteuning J.M. (2024). Resilience following childhood adversity: the need for a heuristic multilevel dynamic framework. Neurosci. Appl..

[bib59] Pischke P.J., Hallman C.J. (2008). Effectiveness of critical event debriefings during operation Iraqi freedom II. United States Army Med. Depart. J..

[bib60] Price R.B., Duman R. (2020). Neuroplasticity in cognitive and psychological mechanisms of depression: an integrative model. Mol. Psychiatr..

[bib61] Prykhodko I. (2021). The psychological readiness model of military personnel to take risks during a combat deployment. BRAIN Broad Res. Artif. Intell. Neurosci..

[bib62] Rapley J. (2017). Embedded mental health: promotion of psychological hygiene within a submarine squadron. Mil. Med..

[bib63] Rushton C.H. (2017). Cultivating moral resilience. AJN Am. J. Nurs..

[bib64] Rybinska Y. (2022). Psycho-emotional state of Ukrainian soldiers before going to the frontline. BRAIN Broad Res. Artif. Intell. Neurosci..

[bib65] Schuhmann C. (2023). How military chaplains strengthen the moral resilience of soldiers and veterans: results from a case studies project in the Netherlands. Pastor. Psychol..

[bib66] Shalev A.Y. (2022). Surviving warfare adversities. A brief survival advice for civilians under war stress. Psychosomat. Med. General Pract..

[bib67] Shelest H. (2024).

[bib68] Stetz M.C. (2011). The effectiveness of technology-enhanced relaxation techniques for military medical warriors. Mil. Med..

[bib69] Stoller C.C. (2012). Effects of sensory-enhanced yoga on symptoms of combat stress in deployed military personnel. Am. J. Occup. Ther..

[bib70] Svetlitzky V. (2020). YaHaLOM: a rapid intervention for acute stress reactions in high-risk occupations. Military Behav. Health.

[bib71] Taylor M.K. (2011). Effect of psychological skills training during military survival school: a randomized, controlled field study. Mil. Med..

[bib72] Timchenko O. (2021). Psychological aspects of captivity in the war in the east of Ukraine. Pol. Psychol. Bull..

[bib73] True P.K., Benway M.W. (1992). Treatment of stress reaction prior to combat using the “BICEPS” model. Mil. Med..

[bib74] Ukraine, A.o.h.i. (2023). Destruction and devastation: one year of russia's assault on ukraine's health care system. https://www.attacksonhealthukraine.org.

[bib75] Ursano R.J., Benedek D.M., Wynn G.H. (2014). Deployment to war and mental health consequences. Lancet Psychiatr..

[bib76] Vermetten E., Jetly R. (2018). Moral decisions in military operations and its relevance for mental health practice and outcomes. Moral Decis Mil. Ment. Health.

[bib77] Vermetten E., Frankova I., Carmi L. (2020).

[bib78] Van Voorhees B.W. (2012). Pilot study of internet-based early intervention for combat-related mental distress. J. Rehabil. Res. Dev..

[bib79] West J.C., Warner C.H. (2019). Fundamentals of Military Medicine.

[bib80] Zabolonyi S., Olytskyi O. (2023). Methods of training future border guard officers shooting with simulation of combat psychological factors. Educol. Discour..

